# Perceptions of document relevance

**DOI:** 10.3389/fpsyg.2014.00612

**Published:** 2014-07-02

**Authors:** Peter Bruza, Vivien Chang

**Affiliations:** Science and Engineering Faculty, Information Systems School, Queensland University of TechnologyBrisbane, QLD, Australia

**Keywords:** document relevance, quantum cognition, information retrieval, cognitive modeling, user modeling

## Abstract

This article presents a study of how humans perceive and judge the relevance of documents. Humans are adept at making reasonably robust and quick decisions about what information is relevant to them, despite the ever increasing complexity and volume of their surrounding information environment. The literature on document relevance has identified various dimensions of relevance (e.g., topicality, novelty, etc.), however little is understood about how these dimensions may interact. We performed a crowdsourced study of how human subjects judge two relevance dimensions in relation to document snippets retrieved from an internet search engine. The order of the judgment was controlled. For those judgments exhibiting an order effect, a *q*–test was performed to determine whether the order effects can be explained by a quantum decision model based on incompatible decision perspectives. Some evidence of incompatibility was found which suggests incompatible decision perspectives is appropriate for explaining interacting dimensions of relevance in such instances.

## 1. Introduction

This article aims to shed light on how humans judge the relevance of documents. We will, however, take a modern view of what a document is. Nowadays individuals and groups interact with one another in a variety of information environments of ever increasing complexity. They are accessing search engines, sharing messages on Facebook, browsing short messages on their mobile devices from microblog sites like Twitter. In this setting, a document is usually very short, e.g., a Twitter post, or in some cases it is not a document at all, but rather a document surrogate, such as the query-biased summaries (snippets) of documents displayed in rankings produced by search engines.

Document relevance has been carefully studied over more than three decades within the fields of information science usually by identifying or employing known inter-subjective dimensions of relevance (Schamber et al., 1990; Barry, 1994; Mizzaro, 1997; Borlund, 2003). For example, Barry and Schamber (1998) identified the dimensions “presentation quality,” “currency,” “reliability,” “verifiability,” “geographic proximity,” “specificity,” “dynamism” and “accessibility” in a comprehensive study. A recent study examined how users determined which list of search results they preferred over another using five dimensions of relevance: “topicality,” “freshness” (currency), “authority” (credibility), “caption quality,” and “diversity” (Kim et al., 2013). Other dimensions have also been identified with respect to a particular genre document. For example, Chu (2012) identified the dimensions “specificity,” “ease of use” and “breadth” in the context of legal documents.

Whilst it is widely accepted that there are a variety of dimensions at play when it comes to judging relevance, little is known of how these dimensions may interact. The aim of this article is to adopt a decision theoretic perspective and test a novel cognitive decision model in which potential interactions between dimensions are a consequence of incompatible decision perspectives which impose an order effect on relevance judgments. Incompatible perspectives are a recent development in a field called “quantum cognition” (See, for example, Conte et al., 2007; Aerts, 2009; Bruza et al., 2009; Pothos and Busemeyer, 2009; Atmanspacher and Filk, 2010; Khrennikov, 2010; Busemeyer et al., 2011; Conte et al., 2011; Trueblood and Busemeyer, 2011; beim Graben et al., 2012; Busemeyer and Bruza, 2012; Conte, 2012; Dzhafarov and Kujala, 2012; Aerts et al., 2013; Blutner et al., 2013; Haven and Khrennikov, 2013). This field aims to apply the formalism of quantum theory in order to more adequately model cognitive phenemona. For example, decades of research have uncovered a whole spectrum of human judgment that deviates substantially from what would be normatively correct according to logic and probability theory. An example of the latter is the following:

Linda is 31 years old, single, outspoken, and very bright. She majored in philosophy. As a student, she was deeply concerned with issues of discrimination and social justice, and also participated in anti-nuclear demonstrationsÓ. Which is more probable:

(a) Linda is a bank teller, or

(b) Linda is a bank teller and is active in the feminist movement?

In this now famous experiment proposed by Tversky and Kahneman (1983), human subjects consistently rate option (b) as more probable than (a). However, according to probability theory, the probability of a conjunction of events must be less than or equal to the probability of a constituent event. Thereofore, according to the axioms of probabilty theory (b) is less probable than (a). Probability judgment errors of this nature have since become known as the “conjunction fallacy.”

**Figure 1:**
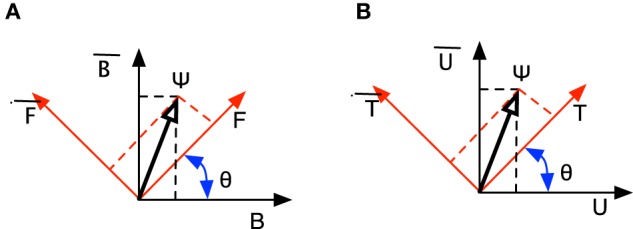
**Incompatible perspectives in a relevance judgment. (A)** Incompatible perspectives in the Linda example. **(B)** Incompatible perspectives in judging document relevance.

The key to explaining the conjunction fallacy using a quantum model is the *incompatibility* between the perspective that Linda is a bank teller and her being a feminist. Consider Figure 1A. The perspective “Linda is a feminist” is represented as a two dimensional vector space where the basis vector *F* corresponds to the decision “Linda is a feminist” and *F* corresponds to “Linda *not* being a feminist.” A similar two dimensional vector space corresponds to the perspective of Linda being a bank teller *B*, or not *B*. Initially, the cognitive state of the subject is represented by the vector Ψ, which is suspended between both sets of basis vectors. This situation represents the subject being undecided about whether Linda is a bank teller or a feminist. Suppose the subject now decides that Linda is a feminist. This decision is modeled by Ψ “collapsing” onto the basis vector labeled *F*. (The probability of the decision corresponds to the square of the length of the projection of the cognitive state Ψ onto the basis vector *F*, denoted ∥**P_F_**ψ∥^2^). Observe how the subject is now necessarily uncertain about Linda being a bank teller because the basis vector *F* is suspended between the two basis vectors *B* and *B* by the angle θ. The hall mark of incompatibility is the state of indecision from one perspective (e.g., the bank teller perspective) when a decision is taken from another (e.g., the feminist perspective). This indecision means the decision maker can't form the joint probability of Linda being both a feminist and a bank teller, Pr(*F, B*) (Busemeyer et al., 2011). (This is crucially different to the situation in standard probability theory in which events are compatible, and thus the joint probability is always defined).

The consequence of incompatibility is an interference term denoted Int. The partial derivation below shows that this term Int appears when the decision of whether Linda is a feminist is made in relation to the incompatible subspace corresponding to the decision perspective of her being a bank teller (represented by projector **P_B_** and its dual **P_B_**^⊥^):

(1) $$p(F)=\;∥{\text{P}}_{\text{F}}\psi {∥}^{2}$$

(2) $$=\;∥({\text{P}}_{\text{F}}\;\cdot \text{I})\psi {∥}^{2}$$

(3) $$=\;∥({\text{P}}_{\text{F}}\cdot ({\text{P}}_{\text{B}}+{\text{P}}_{\text{B}}^{⊥})\psi {∥}^{2}$$

(4) $$=∥{\text{P}}_{\text{F}}{\text{P}}_{\text{B}}\psi {∥}^{2}+\;∥{\text{P}}_{\text{F}}{\text{P}}_{\text{B}}^{⊥}\psi {∥}^{2}+\text{Int}$$

The intuition behind Equation 4 is that the law of total probability is being modified by the interference term. In probability theory this would be expressed as follows: *p*(*F*) = *p(F, B)* + *p(F, B)* + Int. When the interference term is zero, the law of total probability holds. This happens when the decision perspectives are compatible.

Incompatible decision perspectives are a recent development in cognitive modeling and their striking characteristic is the use of “quantum” probabilities. By quantum probabilities, we mean that the decision event space is modeled as a vector space rather than a Boolean algebra of sets. A key differentiator is the use of the interference term. When this term is non-zero, violations of the law of total probability occur. The interference term has been used in models of the perception of gestalt images (Conte et al., 2007; Khrennikov, 2010), models of the conjunction and other decision fallacies (Busemeyer et al., 2011; Conte et al., 2011), modeling violations of rational decison theory (Bordley, 1998; Pothos and Busemeyer, 2009; Khrennikov, 2010), modeling belief dynamics (Trueblood and Busemeyer, 2011) and conceptual processing (Gabora and Aerts, 2002; Gabora et al., 2008; Aerts, 2009; Aerts et al., 2013; Blutner et al., 2013). Broader works relate the formal structures used in quantum theory to cognition and other areas (Bruza et al., 2009; Khrennikov, 2010; Busemeyer and Bruza, 2012; Conte, 2012; Haven and Khrennikov, 2013).

Consider Figure 1B which has the same structure as the Linda problem depicted in Figure 1A. This figure comprises two perspectives regarding a decision of document relevance. Assuming that a human subject perceives a document's relevance via different perspectives in relation to their given information need, the “topicality” perspective is represented as a two dimensional vector space where the basis vector *T* corresponds to the decision “the information is topically related to the information need” and *T* corresponds to “the information is *not* topically related to the information need.” A similar two dimensional vector space corresponds to the perspective of the information being understandable *U*, or not *U*, to the human subject. Initially, the cognitive state of the human subject is represented by the vector Ψ, which is suspended between both sets of basis vectors. This situation represents the subject being undecided about whether the information being perused is topical or understandable. Suppose the subject now decides that the information is topical. This decision is modeled by Ψ “collapsing” onto the basis vector labeled *T*. Once again, the probability of the decision corresponds to the square of the length of the projection of the cognitive state Ψ onto the basis vector *T*, denoted ∥**P**_*T*_ψ∥^2^.

**Figure 2:**
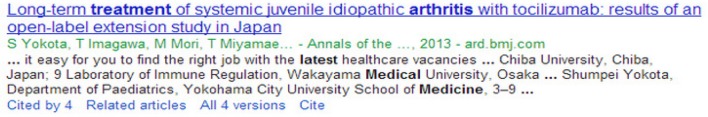
**Example document snippet**.

Observe how the subject is now necessarily uncertain about whether the information is understandable because the basis vector *T* is suspended between the two basis vectors *U* and *U* by the angle θ. The intuition behind incompatibility in this case is that the subject may be confident in deciding the information is topically relevant but remain in two minds about whether they understand the information, for example, if the snippet is interspersed with specialized technical vocabulary as in Figure 2. An important consequence of incompatible decision perspectives is an order effect. In the context of the example, this means the probability of judging that the information is relevant differs when first considering “topicality” followed by “understandability” compared to when these decisions are reversed. This is because when decision perspectives are incompatible, projections do *not* commute, i.e., ∥**P**_*U*_**P**_*T*_ψ∥^2^ ≠ ∥**P**_*T*_**P**_*U*_ψ∥^2^.

The preceding should not be taken to imply that all relevance judgments are modeled in terms of incompatible decision perspectives. In some cases, the perspectives may be compatible. For example, the subject can make a decision that the document is topically relevant and then also be certain in regard to their decision about the document's understandability. In formal terms, compatible decision perspectives entail that the projectors commute, i.e., ∥**P**_*U*_**P**_*T*_ψ∥^2^ = ∥**P**_*T*_**P**_*U*_ψ∥^2^.

The focus is this artice is to explore whether there is evidence for incompatible decision perspectives. The question then becomes how to determine whether the model presented in Figure 1B explains decisions of document relevance. Wang and Busemeyer (2013) have recently proposed an innovative solution to this question. They proved that if there is an order effect and a so called *q*−test holds, then a model based on incompatible decision perspectives like those depicted in Figure 1B is a valid cognitive decision model. In terms of our example, the *q*−test has the following form based on yes(y)/no(n) answers regarding “topicality” and “understandability”:

(5) p(TyUn)+p(TnUy)=p(UyTn)+p(UnTy)

Let *p*_TU_ = *p(TyUn)* + *p(TnUy)* define the probability of different answers when “topicality” *T* is asked first, followed by “understandability” *U*. Conversely, let *p_UT_* = *p(UyTn)* + *p(UnTy)* be the probability of different answers when the order of questions is first “understandability” followed by “topicality.” The *q*−test has the following form:

(6) $$q=p_{AB}-p_{BA}=0$$

The advantage of the *q*−test is that it is a parameter free test. It has successfully been applied to motivate a quantum model in relation to order effects in political survey data (Wang and Busemeyer, 2013). In this article, we will examine: (1) whether there are order effects in relation to decisions pertaining to specific dimensions of relevance, and (2) whether a quantum model based on incompatible decision perspectives explains these order effects.

## 2. Materials and methods

### 2.1. Subjects

Relevance judgments were crowdsourced by the internet based Amazon's Mechanical Turk platform. Crowdsourcing is the outsourcing of tasks to an undefined, large group of people. In the case of Amazon's Mechanical Turk, crowdsourcing is a means of gathering data from users via “human intelligence tasks” (HITs) which are typically surveys for subjects, or “turkers” to answer. Turkers are paid a nominal fee, in this case between 12 and 20 cents per relevance judgment. If the data from the turker is deemed of sufficient quality, the owner of the HIT approves the payment. The quality of the data can be determined automatically by the system whereby after a set period of time, say an hour, then the data will be approved whereby the turker will be paid. This process can also be done manually before and after approval; thus increasing the quality of data collected. In this experiment, the data were manually approved.

The advantages of crowdsourcing is that data can be collected quickly, on a fairly large scale and at a reasonable price. The disadvantage is the extra effort needed in order to safeguard the quality of the data. As Mechanical Turk is internet based, there is little control over who the turkers are, where they are, and indeed, whether they are even human. For example, “bots,” i.e., software programs mimicking humans are known to take part and more or less randomly contribute data to an experiment. As a consequence, the quality of crowdsourced data can vary greatly. To combat this, we purposefully inserted questions in the HITS to collect qualitative data—a technique often used in crowdsoured experiments.

Furthermore, as an additional factor to ensure quality data, both “masters” as well as “normal” turkers were used. Masters have “demonstrated excellence” in performing crowdsourced experiments over an extended period with a required HIT Approval Rate of above 95% over at least one thousand HITs. In contrast to the “masters,” nothing much is known of regarding the performance of “normal” turkers. The experiment was timed to primarily source U.S. based turkers, who are thus likely to be proficient in English, however no tests were conducted to verify English proficiency.

### 2.2. Materials

The materials comprised queries and information in the form of document snippets.

**Figure 3:**
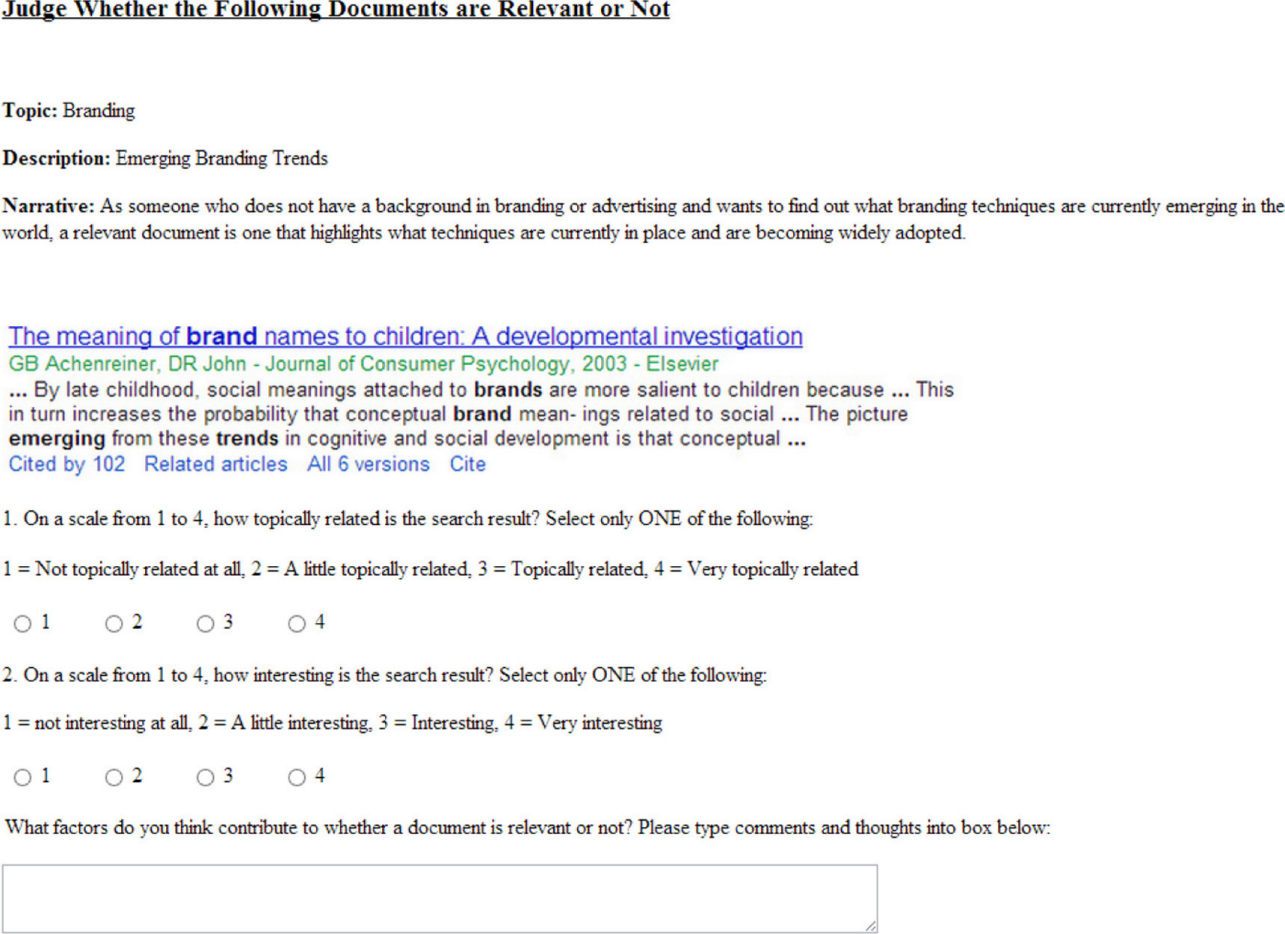
**HIT interface**.

Five queries were developed for this study, each of which is based around an information need, for example, see Figure 3. The query description comprises the name of a query topic, a short description and an accompanying narrative. The narrative is intended to frame the subject's perception of relevance. There is a possibility that the turker's background may interfere with the narrative around the query. For example, if the turker is a fan of technology, then there is significant likelihood that they will be biased toward specific information or brands of technology. The experimenters viewed that bias is intrinsic to search and therefore did not to try to compensate for it (White, 2013). In addition, the background of the turker may hinder their ability to sufficiently engage with the narrative. However there was evidence via the qualitative feedback questions that turkers were able to role-play in a satisfactory way, particularly the “masters.” For example, “..a little hard to determine what this is talking about and if I were a beginner I would have no clue.” or “…makes [the] document highly relevant, since the focus is for emerging technologies in 2013.” Finally, the narrative structure of the queries was adopted from long running Text Retrieval Conference Series run yearly by the U.S. National Institute of Standards and Technology^1^. Each query was designed to collect judgments pertaining to two specific dimensions of relevance chosen by the authors. Table 1 details the titles of the queries and the dimensions of relevance which were studied.

**Table 1:** **Queries and relevance dimensions**.

**Query Title**	**Dimension 1**	**Dimension 2**
Treatment for arthritis	Topicality	Understandability
Emerging branding trends	Topicality	Interest
Emerging technology	Topicality	Credibility
Causes of global warming	Topicality	Believability
Influence of media on the Vietnam war	Topicality	Sentimentality

The relevance dimensions studied are further detailed in Table 2. “Topicality” has been chosen as a primary dimension to be examined across all queries because this dimension has been consistently identified in previous studies as a primary factor in relevance judgments (e.g., Barry and Schamber, 1998; Borlund, 2003; Chu, 2012). In addition, search engine algorithms are based on queries and finding a match in regards to keywords as a matter or correlating topically related material.

**Table 2:** **Definition of relevance dimensions**.

**Relevance dimension**	**Definition**
Topicality	How topically related is the information in the snippet to the query
Credibility	How credible is the information in the snippet
Understandability	How easy is it to understand the information in the snippet
Believability	How believable is the information in the snippet
Interest	How novel/entertaining/interesting is the information in the snippet
Sentimentality	The degree of affective response to the information in the snippet

Secondary dimensions depend on the query. Once the queries had been established, the authors designated likely secondary dimensions. Through pilot studies, the choice for the secondary dimension was refined when other factors began to creep into turker's comments. For example, during initial stages of the pilot, one of the first HITs published was the “Emerging Technology” query involving the dimensions of “topicality” and “understandability.” Very quickly, it was realized that “credibility” was a factor that was constantly brought up by turkers in qualitative feedback. This was possibly also due to the advancement and ubiquity of technology thus rendering “understandability” as not an issue. Other secondary factors were chosen in a similar fashion while some were heavily dependent on the query topic at hand. For example, the topic of global warming is one involving fixed dichotomous positions e.g., people either believe that this is occuring or they don't. Therefore, “believability” seemed likely to be a prominent relevance dimension in this case.

Secondary dimensions that were chosen for study are listed in the column labeled “Dimension 2” of Table 1. “Understandability” was chosen as snippets can sometimes be full of technical jargon, acronyms or specialized terms that can be challenging for the average person to comprehend. The dimension of “Believability” stems from a subject's personal beliefs and biases in relation to the information. A recent study showed that users were subject to their own biases as well as biases inherent in the search engine (White, 2013). “Interest” is the dimension of relevance pertaining to how novel or entertaining the information is. “Sentimentality” is a dimension which pertains to emotional responses to information. Sentiment analysis is a very active area of research in relation to internet-based technologies and applications, for example, data mining techniques to identify positive or negative sentiments or opinions in product reviews.

Corresponding to each query was a query-biased summary of a document, which we will refer to as a document “snippet.” (See Figure 2). Document snippets were used as these are an increasingly prevalent form of information on which decisions of relevance are made in relation to modern information environments. The document snippets used in this study were sourced from the Google search engine.

Snippets were selected based on the likelihood that decisions regarding the two chosen dimensions of relevance were likely to involve some uncertainty. This is because we hypothesize that incompatibility between these dimensions is more likely to occur when such uncertainty was present. Unfortunately, there is no theory to predict which dimensions may be incompatible so a crowsourced pilot study was conducted. This study involved 10 snippets per query with between 8 and 10 master turkers making judgments in each order condition. In order to verify that uncertainty was present a four point rating scale was used to collect decisions. For each query, the snippet for which the *q*− test was closest to zero was selected as being most likely to be subject to incompatibility. None of the subjects in the pilot took part in the experiment presented here. This could easily be verified as each turker has a unique identifier.

### 2.3. Procedure

The experiment (i.e., the HIT) consisted of five elements which were presented in sequence. Each element was based around a query, and a subject was required to process all five elements.

Each element comprises the query description followed by a document snippet, two judgments and finally the input of qualitative data. Figure 3 depicts one such element. In each judgment a subject is asked to rate a dimension of relevance on a four point scale. It was assumed that a subject can make judgments on dimensions within a given query topic independently of other query topics.

A single factor design was employed where the order of the judgments was manipulated. For example, in one condition a given dimension, e.g., “topicality” is rated first (the “non-comparative” context for the decision on topicality), followed by a rating of a the “understability” dimension. In the second condition, the order of the ratings is reversed e.g., the rating on “topicality” is second after the “understandability” dimension is rated (the “comparative” context for the decision on topicality). As each turker has a unique identifier, those turkers who attempted both conditions were removed from the data.

Subsequent to the judgments, subjects were asked to comment on factors that influenced their judgments. This aspect served for both quality control as well as a source of qualitative data to better undertsand the factors involved when turkers make judgments. By doing so, we discarded the data from any turker where the answers were blank, superfluous, e.g., “this is very good and gainful,” or didn't make sense, e.g., “The sway there marketed with different topics.” In the event that qualitative data were borderline acceptable such as “don't know,” or “not sure” (both of which could be supplied by a bot), the time taken to complete the HIT was also taken into consideration: If the time spent was less than 50 s for the HIT, the data were also discarded as we deemed a minimum of 10 s per query as being required to meaningfully read the query topic, rate two dimensions and supply qualitative feedback.

Finally, the Mechanical Turk interface does not afford the ability to time a turker per query, so the time taken to make judgments in relation to a given query could not be collected for analysis.

## 3. Results

A total of fifty “normal” turkers submitted data for the condition where the “topicality” dimension was presented first (non-comparative context for topicality), of which eighteen were discarded. Conversely, thirty-six “normal” turkers submitted data for the comparative context of “topicality,” of which four were discarded. This left *n* = 32 subjects in each condition. Despite repeated attempts to recruit “master” turkers, we failed to secure numbers sufficient for reliable statistical analysis. Therefore, their rating data are not reported but some qualitative responses were retained for illustrative purposes.

**Figure 4:**
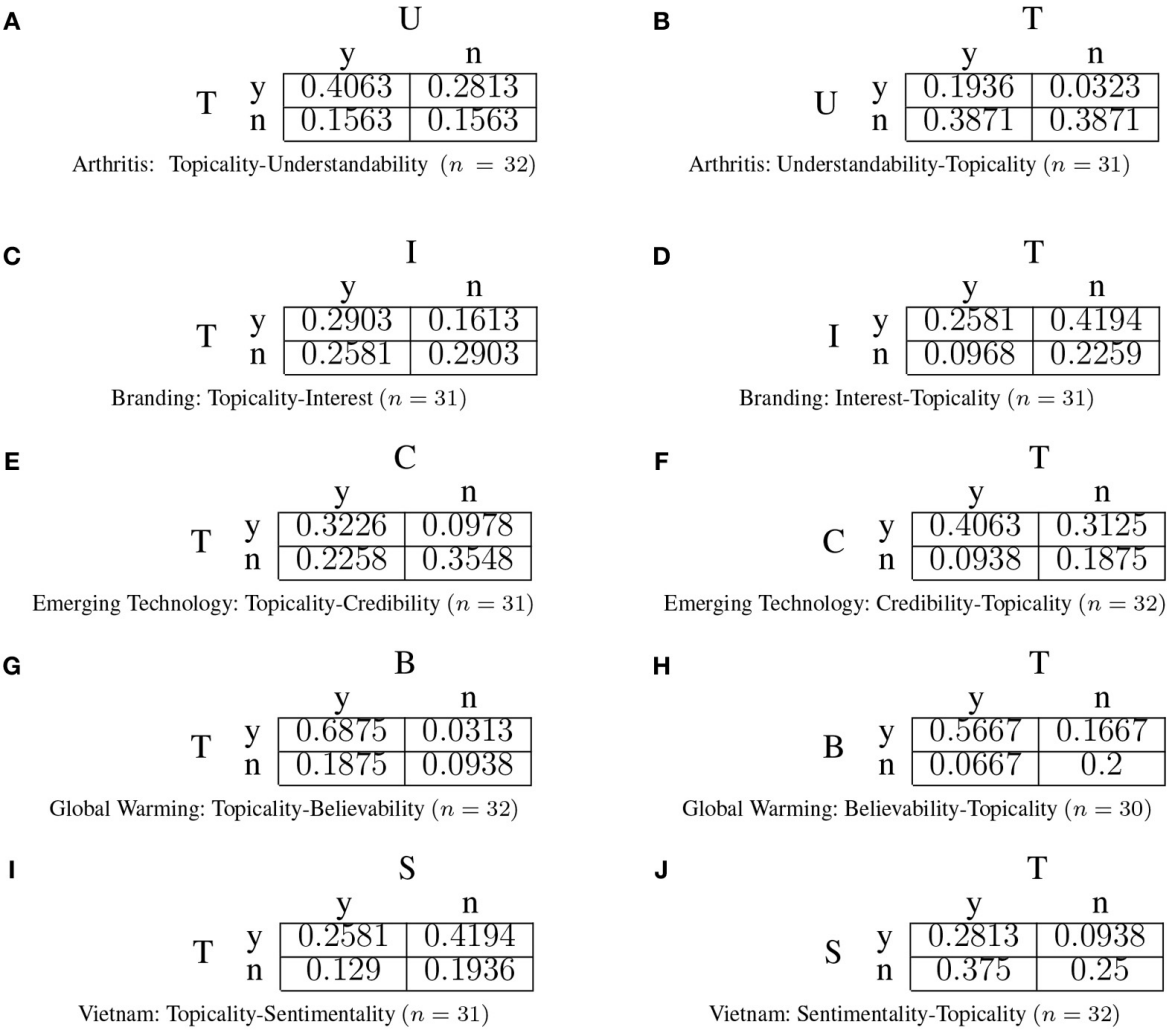
**Yes/no contingency tables from “normal” turkers**. The left hand side represents the condition where topicalilty is decided first (Non-comparative context for a decision on topicality). The right hand side represents the condition where topicality is decided second (Comparative context for a decision on topicality).

The results are presented in yes/no contingency tables in order for the *q*−test to be applied. This was achieved by mapping the four point graded relevance judgments to yes/no decisions in the following way: A grade of 3 or 4 was translated to a “yes,” whereas a grade of 1 or 2 was translated to a “no.” For example, consider Figure 3. Using the proposed mapping, a topical judgment of “4 = Very topically related” and “3 = Topically related” translate into a decision of “yes.” After the yes/no mapping, contingency tables can be constructed for each decision and these are presented in Figure 4 for the “normal” turkers. Some of the queries have data with less than 32 subjects as for these queries a turker rated one dimension, without rating the other. In such cases, the data for that query were omitted.

In order to apply the *q*−test, the presence of an order effect must first be established. An order effect is determined by comparing the agreement rates obtained in a non-comparative vs. a comparative context. An order effect occurs when the proportion of subjects who decided “yes” differs significantly in the comparative vs. non-comparative contexts. A two-tailed χ−square test of equality of proportions between populations was carried out (α = 0.05) and those queries exhibiting an order effect are bolded in Table 3.

**Table 3:** **Summary table of *q*-test values**.

**Query title**	**Dimension 1**	**Dimension 2**	***q***
**Treatment for arthritis**	Topicality	Understandability	0.02^†^
**Emerging branding trends**	Topicality	Interest	0.10
**Emerging technology**	Topicality	Credibility	0.08
**Causes of global warming**	Topicality	Believability	0.01^†^
Influence of media of the Vietnam war	Topicality	Sentimentality	0.08

*Queries with order effect (α = 0.05) are bolded. Queries where *q*-test holds (α = 0.05) are flagged by †*.

Based on the contingency tables presented in Figure 4, the *q*− test values for the “normal” turkers were computed using equation (6) and presented in Table 3.

## 4. Discussion

For the query topics where there is an order effect, the quantum model based on incompatible decision perspectives predicts *q* = 0 (Wang and Busemeyer, 2013). Table 3 accords with this prediction for the queries “Treatment for Arthritis” and “Causes of Global Warming.” However, there are two other queries exhibiting an order effect but for which *q* ≠ 0. In these cases, the prediction of the quantum model may not actualize due to the quite small sample sizes in both conditions, or that the quantum model is not a valid explanation for these queries. More experimentation with larger sample sizes is needed to resolve this distinction.

Four out of five queries displayed an order effect (α = 0.05). The presence of an order effect means that the subjects' decision cannot be validly modeled by a joint probability distribution spanning binary variables corresponding to the underlying dimensions of relevance. For example, consider Figures 4A,B. In the non-comparative context for a decision on topicality, the marginal probability that the document is topical is summed across understandability:

(7) p(T=y)=p(T=y,U=y)+p(T=y,U=n)

(8)               =  0.4063 + 0.2813 

(9)               =0.6876

Note that this probability is significantly different (α = 0.05) when understandability provides the comparative context for deciding topicality: *p*(*T* = *y*) = 0.1936 + 0.3871 = 0.5807. It is this difference which identifies an order effect *but* as the marginal probability is not constant, it is not possible to construct a single joint probability distribution *p*(*T, U*) to model the relevance decisions. As a consequence, a common modeling approach is ruled out. This approach assumes *p*(*T, U*) exists whereby the decision in the non-comparative context around topicality is modeled by the marginal probability *p*(*T*) and the decision in the comparative context is modeled by conditioning the distribution based on how understanding was first decided, i.e., *p*(*T*|*U* = *y*) or *p*(*T*|*U* = *n*).

In summary, order effects were detected between dimensions of relevance for the majority of queries and some evidence that a quantum model based on incompatible decision perspectives is a valid explanation. However, this evidence is not yet strong. Experiments with larger sample sizes and a larger collection of queries and snippets are required to determine the prevalence of incompatible perspectives in perceptions of document relevance. It should be mentioned, however, that this study differentiates itself from many previous studies in that a much larger number of subjects were involved. For example, nine subjects provided relevance judgments in Chu (2012).

According to Cooper (1971) the concept of relevance comprises both “logical relevance” and “utility.” Logical relevance is defined as “whether or not a piece of information is on a subject which has some topical bearing on the information need” and utility has to do with “the ultimate usefulness of the piece of information.” It seems that perceptions of utility or usefulness of a particular snippet involves cognitive processing of a variety of factors including those dimensions examined in this study. It became apparent from the qualitative feedback that relevance is a multifaceted, dynamic decision process. For example, in the “Global Warming” query, “reputation,” “credibility” and “scientific” were used to describe factors that the turkers themselves ranked highly compared to “believability” which was the chosen secondary dimension. This could suggest that the dimensions of “credibility” and “believability” mentioned as being distinct in previous studies are in fact hardly distinguishable during some relevance decisions. Not only were there more than a few factors at play, but the dimensions of “topicality” and “understandability” were featured in qualitative feedback across all queries. Furthermore, comments mentioning multiple (i.e., greater than two) factors were reasonably common. For example, one turker elegantly wrote “whether it (the search result) is on topic, credible, and goes into sufficient detail.” Interestingly, many of these comments noted “topicality” in ways that suggested that even though a snippet was topically related, this did not necessarily translate to the snippet being deemed relevant. This was a shift from the pilot study where turkers would state very clearly in their comments that topicality was nearly always the first factor they considered and if a snippet was topically related, then they would judge it to be relevant. The shift may have been due to the final design in which turkers processed five different queries which exposed them to a broader spectrum of relevance dimensions than was the case in the pilot study. Such qualitative feedback calls the experimental design into question, namely, is it methodologically sound to focus the subjects' attention on two dimensions when more are at play? In addition, were these extra dimensions coming into play because the subject was learning about relevance as they proceeded through the queries? The experimental design did not control for such a learning effect as it was assumed that each query topic could be judged independently of the others. An alternate design would allow subjects to select the two dimensions they deem most prominent and then rate them, or only allow subjects to rate a singe query topic.

## 5. Conclusion

This article put forward an experimental framework for examining whether dimensions of relevance interact via an order effect. The data collected from a crowdsourced study suggests that in some decisions regarding dimensions of relevance, this interaction can be explained in terms of a quantum model based on incompatible decision perspectives. Assuming that such interactions are fairly prevalent, what are the consequences? Currently in information processing systems, such as search engines, there is a general lack of effective user models. Should the user be making decisions of relevenance based on incompatible decision perspectives, then a model of the user based on standard probability would not be appropriate. The field of quantum cognition has shown that incompatibility implies that the law of total probability does not hold. Current computational systems are founded on standard probability theory. For example, consider the corpus-based computational model proposed by Lin and He (2009). This model takes the dimensions of both “topicality” and “sentiment” into account.

At the heart of the model is the following factorization: *p*(**w, z, s**) = *p*(**w| z, s**)*p*(**z, s**), where **w** is a random variable over a vocabulary of terms extracted from the corpus, **z** is a random variable over a set of latent topics, and **s** is a random variable over a set of sentiment labels (e.g., a binary variable describing a positive or negative sentiment). Note at its foundation, the model relies on the joint probability *p*(**z, s**), which describes the joint probability over topics and sentiments. In other words, the model assumes that “topicality” and “sentiment” are *compatible*. Should incompatibility manifest in the user's cognition, such a joint probability is undefined. This opens the door for dissonance between the relevance decisions made by the system as opposed to those made by the user. In short, the presence of incompatible decision perspectives suggests users can better be modeled by a “non-classical” probability theory like that proposed by the field of quantum cognition.

### Conflict of interest statement

The authors declare that the research was conducted in the absence of any commercial or financial relationships that could be construed as a potential conflict of interest.

## Footnotes

^1^http://trec.nist.gov/
